# Remote Application and Use of Real-Time Continuous Glucose Monitoring by Adults with Type 2 Diabetes in a Virtual Diabetes Clinic

**DOI:** 10.1089/dia.2020.0396

**Published:** 2021-01-28

**Authors:** Richard M. Bergenstal, Jennifer E. Layne, Howard Zisser, Robert A. Gabbay, Nathan A. Barleen, Amy Armento Lee, Amit R. Majithia, Christopher G. Parkin, Ronald F. Dixon

**Affiliations:** ^1^ International Diabetes Center at Park Nicollet, HealthPartners Institute, Minneapolis, Minnesota, USA.; ^2^ Onduo, Newton, Massachusetts, USA.; ^3^ Verily Life Sciences, South San Francisco, California, USA.; ^4^ Joslin Diabetes Center, Harvard Medical School, Boston, Massachusetts, USA.; ^5^ University of California San Diego, School of Medicine, La Jolla, California, USA.; ^6^ CGParkin Communications, Inc., Clinical Affairs, Henderson, Nevada, USA.

**Keywords:** HbA1c, CGM, Digital health, Telemedicine, Telehealth, Type 2 diabetes

## Abstract

The Onduo Virtual Diabetes Clinic (VDC) for people with type 2 diabetes (T2D) combines a mobile app, remote lifestyle coaching, connected devices, and live video consultations with board-certified endocrinologists. Adults with T2D (*n* = 594) who were evaluated by a VDC endocrinologist, remotely prescribed and mailed a real-time continuous glucose monitoring (rtCGM) device and used ≥1 sensor completed a CGM satisfaction questionnaire. The CGM satisfaction score was 4.5 **±** 0.8 out of 5. Most respondents (94.7%) agreed/strongly agreed that they were comfortable inserting the sensor remotely and that rtCGM use improved understanding of the impact of eating (97.0%), increased diabetes knowledge (95.7%), and helped improve diabetes control when not wearing the sensor (79.4%). HbA1c (*n* = 372) decreased from 7.7% **±** 1.6% to 7.1% **±** 1.2% (*P* < 0.001; 10.2 months). These data suggest that it is feasible to provide rtCGM directly to individuals with T2D through a VDC without in-office training. Intermittent use of rtCGM was well-received by adults with T2D and was associated with improvement in HbA1c.

## Introduction

Recent clinical trials have demonstrated the benefits of real-time continuous glucose monitoring (rtCGM) for people with insulin-treated type 2 diabetes (T2D).^1–3^ Despite these benefits, adoption of rtCGM in the T2D population has been limited by insurance coverage, the lack of awareness of rtCGM in the primary care setting, where the vast majority of people with T2D are treated, and limited access to diabetes care and education specialists.^4^ New approaches are needed to increase use of rtCGM and advanced technologies for people with T2D.

The Onduo Virtual Diabetes Clinic (VDC) is a comprehensive telehealth program for people with T2D with an innovative model for remotely prescribing and delivering rtCGM for intermittent use without the need for in-office training. The VDC program is designed to support people with T2D and their health care providers between offices. A detailed description of the program has been previously reported.^5,6^ In brief, the VDC program combines a mobile App, remote personalized lifestyle coaching from certified diabetes care and education specialists and health coaches, and connected blood glucose meters and rtCGM devices. The VDC care model includes the availability of live video consultations with board-certified endocrinologists for medication management in addition to prescribing CGM. Participants interact with their care team primarily through the VDC App, which tracks data relevant to participants' diabetes care. Participants are eligible for CGM if they met any of the following criteria at any time during their participation in the program: HbA1c >8.0%, use of insulin or a sulfonylurea, emergency department or urgent care visit in previous six months, no primary care physician (PCP) visit within the prior year, or at the discretion of the VDC endocrinologist. Glucose data are reviewed by the care team and used as a coaching tool in an educational feedback loop that allows participants to associate their glucose levels with their diet, lifestyle, and medication use to optimize self-management.

The objective of this observational survey study was to evaluate VDC program participants' attitudes toward remotely prescribed rtCGM and the relationship between rtCGM use and change in HbA1c.

## Materials and Methods

VDC participants with T2D were included in the analysis if they were clinically evaluated by a VDC endocrinologist, prescribed a rtCGM device, and used ≥1 sensor from March 2018 through July 2019. Depending upon U.S. Food and Drug Administration (FDA) availability, participants were initially mailed either four Dexcom G5 (7-day wear) sensors or three Dexcom G6 (10-day wear) sensors (Dexcom, San Diego, CA), which were provided in one shipment, respectively. The VDC care team provided training videos to help members insert the sensor and onboard to using the rtCGM device. The care team provided education to participants on rtCGM use and encouraged logging of meals, exercise, and medications in the VDC App while using rtCGM. Participants were encouraged but not required to perform the home HbA1c test. Intermittent sensor wear schedules were individualized based upon interactions with the care team and participant preferences. Participants were sent a CGM satisfaction questionnaire electronically via the VDC App. This analysis was approved by the Western Institutional Review Board.

### Outcomes

#### CGM awareness and satisfaction

Participants were asked if they were familiar with CGM or had heard of CGM before joining the VDC program with yes or no response options. Participant satisfaction with rtCGM use was assessed using a subset of nine questions from the validated 44-item CGM Satisfaction Scale.^7^ Questions were selected to assess three CGM-related themes: educational value, impact on diabetes self-management, and device satisfaction. Response to each item was assessed on a 5-point Likert scale: 1 = strongly disagree, 5 = strongly agree.

#### Change in HbA1c

Change in HbA1c was evaluated as the difference between the most recent HbA1c level obtained before using the first sensor (baseline) and the most recent HbA1c ≥90 days after the baseline sensor wear (follow-up). HbA1c data were obtained from a central laboratory analyzing the participant home-test samples or verified with the primary care physician by the health coach or obtained directly from the provider or medical record. HbA1c testing is requested, but not required for program participation, thus data were only available for a subset of participants.

### Statistical analysis

Baseline characteristics of survey respondents and nonrespondents were summarized and compared. All subsequent analyses were performed only for survey respondents. Overall mean satisfaction score was calculated based upon the sum of responses to the nine satisfaction-related questions with reverse scoring applied to the negatively worded question (“CGM sometimes gives me too much information to work with.”). Mean score for each question was also summarized. All glycemic outcomes were evaluated by two-tailed *t*-tests using R software.^8^ Statistical significance was defined as *P* < 0.05.

## Results

A total of 761 participants were sent the satisfaction survey and 594 (78.1%) responded. Baseline demographic and clinical characteristics of survey respondents and nonrespondents are presented in Table 1.

**Table 1.** Participant Characteristics

Variable	Survey respondents (*n* = 594)	Nonrespondents (*n* = 168)	*P*
Age, year	53.0 ± 8.4	48.5 ± 9.4	<0.001
Female, *n* (%)	370 (62.3)	107 (64.1)	0.74
Body mass index	35.4 ± 7.7^a^	36.1 ± 7.4^b^	0.28
Baseline HbA1c, (%)	7.7 ± 1.6^c^	8.0 ± 1.7^d^	0.035
Medication use, *n* (%)			
Sulfonylurea	153 (25.8)	35 (21.0)	0.24
Insulin	217 (36.5)	63 (37.7)	0.84
Geography, *n* (%)			0.99
Urban	425 (72.5)^e^	119 (72.1)^f^	
Rural	161 (27.5)^e^	46 (27.9)^f^	

Data are presented as mean ± standard deviation unless otherwise indicated.

^a^ *n* = 550.

^b^ *n* = 147.

^c^ *n* = 563.

^d^ *n* = 148.

^e^ Out of a total *n* = 586.

^f^ Out of a total *n* = 165.

### Sensor use

Of the survey respondents, 279 (47.0%) participants received the G5 sensor, 290 (48.8%) received G6, and 25 (4.2%) received both types of sensors. Mean days of sensor wear was 31.1 ± 26.5 days intermittently over a period of 4.8 ± 3.2 months. The intermittent sensor wear schedule was individualized, and timing varied based upon care team recommendations and participant preferences.

### rtCGM awareness and satisfaction

Among the 594 survey respondents, 60.8% indicated that they were not familiar with or had not heard of CGM before participation in the VDC program.

The mean overall rtCGM satisfaction score was 4.5 out of 5. The majority of respondents indicated agreement or strong agreement that rtCGM use increased their diabetes knowledge; improved their understanding of medication importance; made it easier to perform other self-management behaviors; improved their understanding of how food impacts their diabetes control; increased their understanding of how everyday tasks impact their diabetes; and helped improve their diabetes control when not wearing the rtCGM device. A majority of respondents agreed or strongly agreed that they felt comfortable inserting the rtCGM sensor (94.7%) and 88.4% indicated that they would like to use rtCGM again. Most respondents, 70.5%, disagreed or strongly disagreed that CGM provided too much data.

No differences in overall satisfaction were observed for insulin users (*n* = 217) compared to noninsulin users (*n* = 377) (*P* = 0.77), participants meeting the American Diabetes Association (ADA) treatment of HbA1c <7.0% (*n* = 209), or participants above target (*n* = 354) (*P* = 0.07). There were also no differences between Dexcom G5 (*n* = 279) and G6 (*n* = 290) users (*P* = 0.32).

### Change in HbA1c

**FIG. 1.**
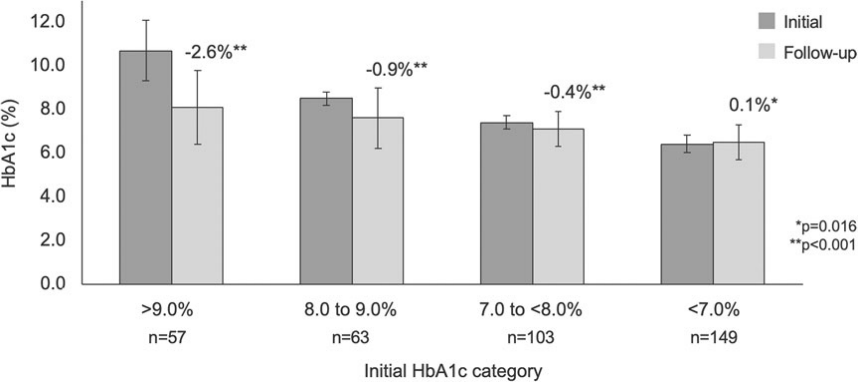
Change in HbA1c stratified by baseline HbA1c.

The mean follow-up time-period for HbA1c measurement (*n* = 372) was 10.2 ± 4.0 months. The mean time-period from the initial sensor wear to the follow-up HbA1c measurement was 8.3 ± 3.8 months. Significant reductions in HbA1c from baseline were observed within the total respondent cohort, from 7.7% ± 1.6% to 7.1% ± 1.2%, change −0.6% ± 1.5%, *P* < 0.001. There was no difference in the overall change in HbA1c between survey respondents and non-respondents (*n* = 54). Change in HbA1c stratified by baseline categories of >9.0%, 8.0% to 9.0%, and 7.0% to <8.0% is presented in Figure 1.

The percentage of participants achieving Health Effectiveness Data Information Set (HEDIS) HbA1c treatment target (HbA1c <8.0%) increased from 46.0% at baseline to 65.3% at follow-up in the insulin user group and from 78.6% at baseline to 93.1% at follow-up in the noninsulin user group. Significant reductions in HbA1c were observed at follow-up in both the insulin and noninsulin user groups with a baseline HbA1c ≥8.0%, −1.5% ± 2.1% and −2.0% ± 1.7%, respectively (both *P* < 0.001). In addition, a significant reduction in HbA1c was observed in participants with <30 days sensor wear (median wear time) and ≥30 days of sensor wear, −1.3% ± 1.8% and −2.1% ± 1.9%, respectively (both *P* < 0.001), the group of participants with baseline HbA1c ≥8.0%.

## Discussion

This study demonstrated that through the Onduo VDC, it is feasible to remotely prescribe and mail rtCGM devices to people with T2D and for participants to successfully insert sensors and use rtCGM without in-office training. This may extend use of CGM to the primary care setting where there is often limited diabetes educator support. Intermittent use of CGM was well-received by VDC participants who reported high satisfaction with rtCGM as an educational tool that positively influenced their perception of diabetes self-management. The use of rtCGM was associated with a significant improvement in HbA1c at 10 months in those not meeting the ADA treatment target, independent of insulin use. In addition, there was a large shift in the percentage of participants meeting the HEDIS HbA1c target of <8.0% at follow-up; this may have important clinical and economic implications. To our knowledge this is the first study to report findings of user acceptance and clinical outcomes associated with CGM use in a virtual diabetes management program.

Participants reported that rtCGM use increased their knowledge about the impact of food, medication use, and everyday activities on their glucose levels. It appears that rtCGM influenced participants' health behaviors as evidenced by improvements in glycemic control. The finding that nearly 80% of participants reported that rtCGM helped them improve their diabetes control even when not wearing their device suggests a possible beneficial carryover effect. Moreover, nearly 90% of participants reported that they would like to use rtCGM in the future if made available. It was interesting to note that individuals who were already meeting the ADA treatment target for HbA1c reported similar overall satisfaction with rtCGM use compared with those with higher HbA1c levels.

While persistent daily use of rtCGM is considered essential for intensive insulin management,^1–3,9–14^ clinical trials and recent meta-analyses have reported the value of rtCGM use in individuals with T2D independent of their treatment regimen.^15–18^ The real-world data included in this report add to the growing body of evidence indicating that intermittent rtCGM use with less intensive treatment regimens is associated with significant glycemic improvements,^19–21^ creates valuable teaching opportunities,^22^ and is effective in promoting desired self-care behaviors.^20^

Our study has some limitations. First, although the items included in our survey were derived from a validated assessment instrument,^7^ we used a subset of these questions in our survey, which may not fully represent participant experience with other aspects of rtCGM use. Second, there may have been response bias in this study: those who responded to the survey were older and had lower baseline HbA1c levels. In addition, respondents and non-respondents who elected to provide follow-up HbA1c (which was not required) may have differed from those who did not provide follow-up data. Third, in this real-world observational study, there was no control group. Engagement and program interaction and the impact on outcomes were not evaluated in this analysis but is currently being evaluated in an ongoing investigation. In addition, further investigation is needed to determine the cadence of intermittent rtCGM use and stepped coaching in the VDC care model. Analyses of other rtCGM metrics (e.g., time in range, time above range, and glucose management indicator) are underway in ongoing studies and these results should further elucidate the impact and potential benefits of rtCGM use in adults with T2D.

## Conclusion

Remote prescription of rtCGM for intermittent use as part of the overall VDC care model was well-received by participants with T2D, the majority of whom indicated that they were comfortable inserting their sensors at home. Overall, the results of this study suggest that intermittent use of rtCGM was a beneficial diabetes management tool that increased participants' knowledge about the impact and importance of diabetes care behaviors, enhanced engagement in their self-management regimens, and was associated with improvements in glycemic control. Access to rtCGM and specialist care through the VDC program is a novel approach to support people with T2D managed in primary care.

## Authors' Contributions

R.M.B., J.E.L., H.Z., R.A.G., N.A.B., and R.F.D., contributed to the concept and design of the study. N.A.B, R.M.B., J.E.L., H.Z., R.F.D., and C.G.P. contributed to the acquisition, analysis, and interpretation of the data. N.A.B and A.A.L. contributed to statistical analyses. J.E.L. and C.G.P. wrote the first draft of the article. All authors contributed to critical revision of the article for important intellectual content. R.M.B. had full access to all of the data in the study and takes responsibility for the integrity of the data and the accuracy of the data analysis.
